# The Antifungal Effect of Pyroligneous Acid on the Phytopathogenic Fungus *Botrytis cinerea*

**DOI:** 10.3390/ijms24043080

**Published:** 2023-02-04

**Authors:** Giorgia Pertile, Magdalena Frąc

**Affiliations:** Institute of Agrophysics, Polish Academy of Sciences, Doświadczalna 4, 20-290 Lublin, Poland

**Keywords:** strawberry pathogen, wood vinegar, inhibition test, metabolic profile, MT2 microplate

## Abstract

In recent years, climate change has intensified harsh periods of rain alternating with periods of drought, leading to an increase in the presence of phytopathogenic fungi. In this study, we want to analyse the antifungal properties of pyroligneous acid against the fungal phytopathogen *Botrytis cinerea*. Through the inhibition test, we observed that the application of different dilutions of pyroligneous acid rarefied the growth of the fungal mycelium. Furthermore, we have seen through the metabolic profile that *B. cinerea* is not able to use pyroligneous acid as a resource or even grow in close contact with this resource. Moreover, we observed that the pre-incubation of the fungus in pyroligneous acid leads to a reduction in biomass production. These results give us hope for the possible use of this natural substance as a possible substance to protect plantations from pathogen attacks.

## 1. Introduction

In recent years, due to global warming, pollution, and climate change, significant changes in agriculture, leading to negative economic (lower production yields), qualitative (lower quality and durability of the product on the food market) and health-related (for humans but also for the environment) results, are observed [1,2]. For this reason, in 2020, the European Commission (EC) decided to create a group of policy initiatives called the European Green Deal [2,3]. These initiatives aim to be net-zero by 2050, achieving part of the goal in 2030 by reducing emissions by at least 55%. Unfortunately, agriculture is responsible for 24% of greenhouse gas emissions, especially in the emission of 60% of N_2_O and 50% of CH_4_ [3], which is why these initiatives can restore nature and recover biodiversity in such a way as to promote a healthy and efficient ecosystem that can be more resilient for future climate change, including absorption and storage of carbon. An environmental system is sustainable when it does not have a strong negative impact on the environment, helps to mitigate climate change, does not result in the loss of biodiversity, and guarantees food security, nutrition, and public health [2,3].

Poland is among the European countries with the largest production of strawberries, reaching a collection area of 39.46%, a yield of 38.34%, and production of 15.13% compared with the 27 countries belonging to the European Union [4]. However, the presence of plant pathogens can affect production by affecting the health of the plant, with a consequent reduction in fruit production [5,6,7]. Therefore, the search for appropriate methods to improve strawberry production and ensure the reduction or elimination of fungal pathogens in crops is one of the most important challenges for modern, sustainable, and precise agriculture. Finding the right substances and compounds to which fungal pathogens show sensitivity is important both from an economic point of view (production volume and efficiency) and from the point of view of guaranteeing plantation health and soil quality [8].

*Botrytis cinerea* has a necrotrophic lifestyle, which means that infecting the plant leads to the death of the host tissues, and subsequently, the fungus survives the saprophytic stage on the necrotic tissue or produces long-lasting structures, called sclerotia [9,10]. It infects plants in all climatic areas, especially the aerial part of the plant, in the pre- and post-harvest phases, and can remain in a quiescent state in host tissues for a long time. This plant pathogenic fungus can infect plants in the nursery, ornamental plants, field crops, and orchards [11,12,13]. Its infection of a large sector of plants and the extreme use of fungicides in recent years have led to the development of genes that give resistance to pesticides [9,11,14,15]. The factors that can influence the spread of the disease (such as high production of conidia, drops of water on the surface, presence of nutrients) are very important and must be taken into consideration in order to control the spread of infections by *Botrytis cinerea* [9,14,16,17]. Another factor that makes this fungus important in both the agricultural and economic fields is that the mycelium is active at moderate temperatures, but can also be active at temperatures close to zero, thus being able to infect fruit during the storage phase [9,17,18].

Pyroligneous acid (PA) is a by-product of the pyrolysis of plant biomass or organic material under anaerobic conditions or limited oxygen supply, leading to the production of biochar, volatile materials (as pyroligneous acid and tar), and various gases (H_2_O, CH_4_, H_2_, CO_2_, and CO) [19]. The volatile materials can be channelled into a cold tube to condense the substance and obtain pyroligneous acid. For this reason, we can consider the production of this natural substance as an aid for the reduction of greenhouse gas emission as well as being a carbon sink [19,20]. This natural substance presents itself with its classic amber-brown colour and a smoky smell. Pyroligneous acid is formed by various substances obtained from the depolymerisation of three polymers (cellulose, hemicellulose, and lignin) derived from plant biomass [19]. The various substances that make up PA include more than 200 organic compounds, including organic acids, alkanes, phenols, alcohols, and esters [19,20,21,22]. The most abundant substance (about 50%) is acetic acid, which gives PA its characteristic pH below 3, while the phenolic group gives it a smoky smell [19]. Recently, researchers have begun to study the possible uses of PA in agriculture, highlighting an increase in yield, product quality, and plant health [23,24], improvement of germination and seed growth [25,26,27], resistance to fungal pathogens [28,29], resistance to exposure to high concentrations of ozone [30], an increase in nitrogen concentration in the soil [31], and resistance to stress, disease, and insects [32]. PA inhibits the growth of fungal mycelium due to its negative effects on the speed of division, cell membranes, electrolytes, and protein synthesis [28]. PA has all the positive characteristics that could make it a future substitute for the use of chemical pesticides, one of the key points of the European Green Deal. In fact, the presence of phenols, esters, and acetic acid provides antioxidant and antimicrobial properties, while phenolic compounds and furaldehydes are associated with antifungal properties [19]. Our interest in testing PA as a possible antifungal against *B. cinerea* was born from the fact that other researchers have already identified these antifungal properties against many fungi, including *Pythium*, *Penicillium*, *Rhizobium*, *Sclerotia*, and *Fusarium*, and, in particular, it has been proven that this natural substance inhibits the growth of the mycelium of *Phytophthora capsici* and *Verticillium dahliae*, which are also fungal pathogens of the strawberry plant [22,33,34,35].

The aim of this research was to test whether PA has a negative effect on the growth of the phytopathogen *Botrytis cinerea*. Many studies to prove this effect of PA have used the inhibition test [36,37]. In addition to this method, we propose the analyses that are part of the Biolog Phenotype Microarray (PM) technique. Through these analyses, we can measure the use of pyroligneous acid (MT2) or different carbon sources (FF) and production of fungal biomass against different types of substances by studying the metabolism of organisms. In this article, we analyse the susceptibility/resistance of *Botrytis cinerea* in three ways: (1) Through the inhibition test, we focused on the efficiency of the application of different concentrations of pyroligneous acid on the growth of the fungal mycelium. (2) Through the MT2 microplate (Biolog™), we observed how pyroligneous acid can influence the incubation of the fungal mycelium from the point of view of the use of the analysed substance as a resource. (3) Through the FF microplate (Biolog™), we observed whether pre-incubation for 5 days with different concentrations of pyroligneous acid can affect the Average Well Colour Development (AWCD), the Average Well Density Development (AWDD), and the substrate richness (R) of *B. cinerea*.

## 2. Results

The identification of three selected strains of *Botrytis cinerea* was confirmed through the polymerase change reaction analysis with a specific primer (Figure S1).

### 2.1. Effect of Pyroligneous Acid on Fungal Mycelium Growth on Petri Dishes

**Figure 1:**
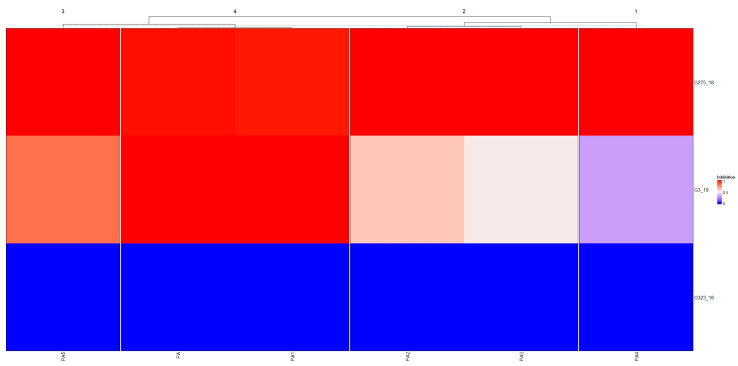
Inhibition profile of *Botrytis cinerea*. Colour scale of the heatmap indicates the inhibition degree (red represents stronger inhibition and blue represents lower inhibition). Abbreviations: PA is PA pure; PA1 is PA 1:2; PA2 is PA 1:8; PA3 is PA 1:16; PA4 is PA 1:32; and PA5 is PA 1:36.

The inhibition test confirmed the negative effect of PA on the fungal mycelium growth. During the 5 days of the incubation, we observed a change in the structure of the mycelium (Figure S2). Through comparison of all of the PA treatments versus the control (PA 0), it was observed that the mycelium grew in a rarefied way, not fully covering the culture surface. Furthermore, it was possible to observe how the mycelium in contact with PA 0 presented the classic soft structure, while with the other concentrations of PA, a thin mycelium with an almost dry appearance was observed. Analysis of the inhibition areas measurements (Figure 1) indicated that the three strains of *B. cinerea* reacted differently to tested PA treatments, highlighting different degrees of sensitivity: strong, medium, and low. Observing the treatments applied, we were able to see, as a function of the cluster analysis, the formation of three groups.

There was a clear division into two groups according to the intensity of the inhibition towards *B. cinerea*. Pure pyroligneous acid and the 1:2 and 1:36 dilutions greatly influenced the development of the fungal mycelium, while the other three dilutions (1:8, 1:16, and 1:32) had a medium effect on the production of the fungal biomass. Through this preliminary analysis, we were able to ascertain that direct contact of the mycelium with pyroligneous acid leads to an inhibition of the growth of the mycelium, confirming the antifungal properties against *B. cinerea* of this tested natural substance. This first analysis indicated the effect of pyroligneous acid on the growth of the fungal mycelium, especially since a visual result of the antifungal aspect of natural PA was obtained.

### 2.2. Effect of Pyroligneous Acid on Growth of Botrytis cinerea in MT2 Plates

**Figure 2:**
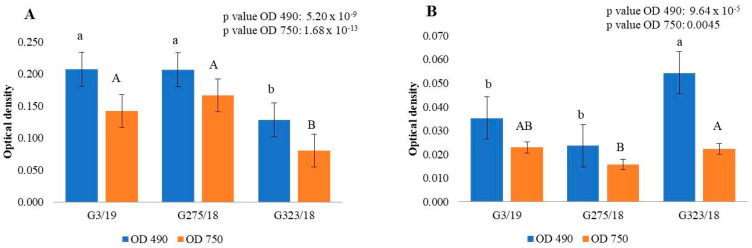
Optical density values from the MT2 microplates for the three different strains of *B. cinerea* in function of FF inoculating fluid (**A**) and PDB medium (**B**). Each value is represented with the standard error. The lower-case letters (referring to the 490 nm optical density) and the upper case letters (for the 750 nm optical density) represent the results obtained after applying the post hoc Wilcox test (Tables S1, S2, S4, S5, S7, S8, S10 and S11).

**Figure 3:**
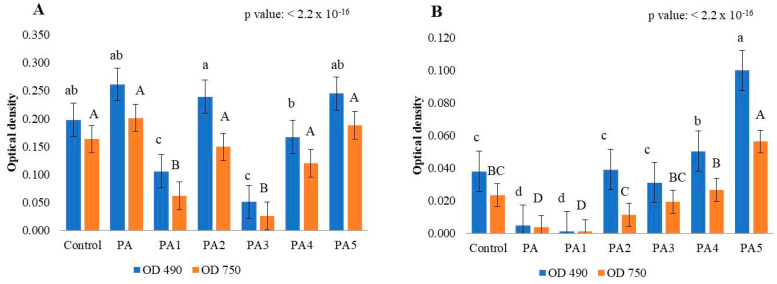
Optical density values from the MT2 microplates for the seven different analysed treatment of pyroligneous acid (PA) in function of FF inoculation fluid (**A**) and PDB medium (**B**). Each value is represented with the standard error. The lower-case letters (referring to the 490 nm optical density) and the upper case letters (for the 750 nm optical density) represent the results obtained after applying the post hoc Wilcox test (Tables S1, S3, S4, S6, S7, S9, S10 and S12). Abbreviation: Control is without adding PA; PA is PA pure; PA1 is PA 1:2; PA2 is PA 1:8; PA3 is PA 1:16; PA4 is PA 1:32; and PA5 is PA 1:36.

The results of the MT2 plates indicated that *B. cinerea* can use PA as a carbon source and simultaneously produce fungal mycelium. Through this analysis, we first identified that if the fungus was incubated in FF inoculating fluid or PDB medium, it had a completely different response to exposure to different dilutions of pyroligneous acid. Analysing the optical density at 490 nm (Figure S3), we were able to observe a division of the treatments performed in the FF inoculating fluid and PDB medium into three groups. All treatments with low fungal respiratory activity were placed in groups I and II, in PDB medium and FF inoculum, respectively. All treatments with high fungal respiration activity were placed in group III, in FF inoculum. The same results were observed for biomass production measured via the 750 nm optical density (Figure S4). The results indicated that the fungus *B. cinerea* pre-incubated in the PDB medium with PA showed a lower utilization and growth than the fungus incubated in the PDA medium with PA and analysed in the FF inoculating fluid. The inhibitory capacity of pyroligneous acid towards the phytopathogenic fungus *B. cinerea* indicated statistically significant differences (Figure 2 and Figure 3). Analysing the fungal strains, we observed how the reaction of the fungus changed, depending on the inoculum liquid during the analysis. In particular, we observed how the G323/18 strain incubated in FF inoculum had a low value of biomass utilization and production compared to that observed in PDB medium, and that the other two analysed fungal strains showed behaviour in both liquids that was completely opposite to the strain described above (Figure 2). Regarding the ability of the plant pathogenic fungus *B. cinerea* to decompose this natural substance (Figure 3), we observed a strong inhibition by the treatments PA 1:2 (PA1) and PA 1:16 (PA3) in FF inoculum, and pure PA (PA) and PA 1:2 (PA1) in PDB medium. Especially, we noticed how the values of utilization and production of fungal biomass increased with the increase in the values of the dilutions of pyroligneous acid in PDB medium (Figure 3B). The analysis of the ability of *Botrytis cinerea* to decompose pyroligneous acid confirmed that the low dilutions of pyroligneous acid induce an inhibition not only in the biomass production phase, but also during the respiration of the fungus, because, during exposure to low dilutions, we observed a low ability of the fungus to use pyroligneous acid as a resource. By grouping the results obtained from the analysis of the MT2 microplates into four different groups (fast, fast-medium, slow-medium, and slow), it was shown that pyroligneous acid is used, and the fungus can produce biomass quickly, only when the fungus is incubated in FF inoculum (see Table S13). By calculating the rate 490/750, the metabolism efficiency of the tested fungus was evaluated. The measurement of the optical density at 490 nm shows the utilization of the substrate, and at 750 nm indicates the production of fungal biomass. A low value of this ratio means that the fungus has an efficient metabolism; therefore, a high development of fungal biomass is accompanied by a low use of the substrate. The opposite situation, where a low production of biomass is accompanied by a high use of substrates, indicates a situation of stress at the metabolic level [38]. The rate 490/750 obtained from the MT2 microplates indicated that pure pyroligneous acid and its analysed dilutions put the metabolism of *B. cinerea* in a state of stress. Moreover, comparison of three tested fungal strains indicated that only the strains G3/19 and G275/18 had an efficient metabolism in the dilutions 1:16, 1:32, and 1:36, while the strain G3/19 incubated in FF inoculum exhibited good metabolism after incubation in pure pyroligneous acid (Table S13). In conclusion, we can state that dilutions of pyroligneous acid lower than 1:36 can lead to the complete inhibition of the growth of the fungal mycelium of *Botrytis cinerea*.

### 2.3. Metabolic Profile of Botrytis cinerea Pre-Cultivated on Pyroligneous Acid

**Figure 4:**
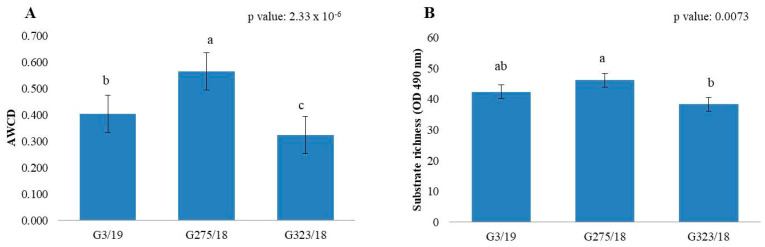
AWCD (**A**) and substance richness (**B**) indices for the three different strains of *Botrytis cinerea*. Each value is represented with the standard error. The lower-case letters represent the results obtained after applying the post hoc Wilcox test (Tables S14–S18).

**Figure 5:**
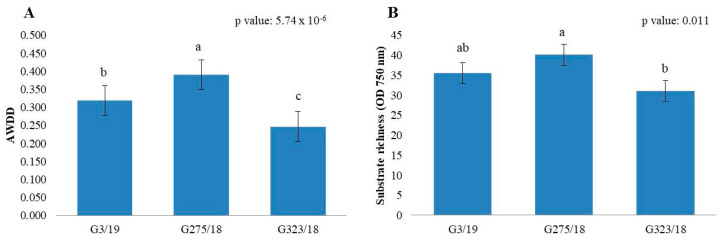
AWDD (**A**) and substance richness (**B**) indices for the three different strains of *Botrytis cinerea*. Each value is represented with the standard error. The lowercase letters represent the results obtained after applying the post hoc Wilcox test (Tables S19–S23).

The metabolic profile of tested fungal strains indicated how the pre-incubation of each *Botrytis cinerea* strain in the presence of different dilutions of pyroligneous acid can affect the use of carbon substrates and biomass production on 95 different carbon sources. The AWCD and AWDD indices were statistically influenced by the fungal strains analysed (Tables S14 and S19), demonstrating a clear difference in the metabolic profile between the fungal strains (Figure 4 and Figure 5). Furthermore, analysis of the index of the richness of the substrate at 490 nm and 750 nm indicated that, in both optical densities, significant differences between the fungal strains were observed. AWDD analysis showed that the tested treatments were not significantly different (*p* value 0.05). Applying the post hoc test, we can observe a difference between the control (pre-incubated fungus without pyroligneous acid) and the highest dilution of pyroligneous acid (PA:1600). This indicates that the application of this dilution results in a decrease in fungal biomass production within the 95 different carbon resources.

## 3. Discussion

The results obtained through this research indicated that the application of pyroligneous acid has a negative effect on fungal mycelium growth and metabolism. Many researchers have begun to test this natural substance, identified as antibacterial and antifungal due to the molecules it contains. Wei et al. [22] tested the pyroligneous acid from walnut branches against various plant pathogens, such as *Phytophthora capsici*, *Colletotrichum orbiculare*, *Valsa mali*, *Cochliobolus sativus*, *Helminthosporium sativum*, and *P. infestan*, observing a clear inhibition of these pathogens incubated in different types of pyroligneous acid. Jung [35] has already tested the effect of pyroligneous acid on *Botrytis cinerea* and observed a total growth inhibition for the 1:2 and 1:4 dilutions, showing a slight growth of the mycelium in the 1:8, 1:16, and 1:32 dilutions. Chen et al. [39] tested pyroligneous acid from *Eucommia ulmoides* against *Botrytis cinerea* and found that the acid, even at dilutions lower than 1:200, can have a strong impact on the growth of the gray mold fungal mycelium. Moreover, the results of our research indicated that 1:1600 dilution of PA affected metabolic profiles of *B. cinerea*, by inhibiting metabolic biodiversity indicators. However, there is a need to deepen knowledge concerning particula carbon sources and organic compound to select the most effective in supporting PA in efficient control of phytopathogens. These different results can be justified by the fact that the quantity and type of molecules characterizing pyroligneous acid depend on the origin of the biomass [19,40]. This is the first article describing the use of MT2 microplates to study the possible inhibitory effects of pyroligneous acid on pathogenic fungi. This methodology is widely used to characterize and monitor metabolism (respiration and biomass production) to quickly underline and estimate the sensitivity of the microorganism to this substance [10]. In other studies, the MT2 plate methodology was applied to identify the utilization of different carbon resources after pre-incubation in different *Petriella setifera* litter [41,42], and to validate an effective methodology to detect resistance or susceptibility of *Fusarium* sp. To fungicides [43]. These recent works are proof of the use of this methodology to identify the sensitivity or resistance of fungi to exposure to different substances. From the results obtained from the MT2 microplates, we observe that the phytopathogenic fungus *Botrytis cinerea* is unable to use pyroligneous acid as a resource, and that the incubation of the fungus with this analysed natural substance led to difficult growth, underlining the properties of this substance in inhibiting, or even blocking, the production of fungal biomass. Through these results, we can consider MT2 microplates as a good approach to understand and observe whether a substance can inhibit fungal growth, as it was previously proposed by Frąc et al. [43], and therefore identify those substances that can interfere in the metabolism of pathogenic fungi. From the point of view of the metabolism profile, we observe how the different dilutions of pyroligneous acid do not greatly influence the functional diversity (calculated through the AWCD, AWDD, and R indices), but rather are influenced by the individual metabolisms of each strain. For the AWDD index, we observed that the analysed treatments present a “slight” non-significant difference (*p* value 0.0507, Figure S5). This indicates that the application of the dilutions of pyroligneous acid negatively interferes with the production of the fungal biomass of *Botrytis cinerea*, also confirming the results obtained from the MT2 plates, underlining how pyroligneous acid negatively affects the production of fungal biomass. Jung [37] tested different concentrations of pyroligneous acid and found that dilutions lower than 1:4 inhibited the growth of *Botrytis cinerea*, while Chen et al. [39] noted that the pyroligneous acid had an obvious and strong effect on the pathogenic fungus for the dilutions to 1:200 and 1:400, respectively. The fact that, in our study, we found that 0.0625% *v*/*v* presents a strong inhibition on biomass production, compared to the other two dilutions, could be connected to the fact that the pyroligneous acid in this dilution was less present and that a large amount of water aided its absorption by *Botrytis cinerea*. We understand that a very low percentage of pyroligneous acid is enough to alter the fungal metabolism, and therefore consequently manifests in the slow growth of the mycelium.

## 4. Materials and Methods

The design of the research included some steps involving the molecular identification of fungal strains selected for the study, determination of fungal strain sensitivity to different concentrations of pyroligneous acid using the paper disc plate method and MT2 microplates method to select the most effective concentration in the control of *B. cinerea*. Moreover, the influences of the most effective concentrations of PA on metabolic profiles of *B. cinerea* were tested. 

### 4.1. Fungal Strains

Three different strains of *Botrytis cinerea* (G275/18, G323/18, and G3/19) isolated from infected strawberry plants were tested within this research. The selected strains came from the microorganism collection of the Laboratory of Molecular and Environmental Microbiology, Institute of Agrophysics, Polish Academy of Sciences (Lublin, Poland) and were isolated as a part of a project Biostrateg (BIO-STRATEG3/344433/16/NCBR/2018). To confirm that these three strains belong to the species *B. cinerea*, a specific PCR analysis with two specific primers for the detection of the fungal pathogen *B. cinerea* (Patent number P.431989) was performed. 

### 4.2. Pyroligneous Acid

The pyroligneous acid (PA) (product number W296708, SIGMA-ALDRICH, Saint Louis, MO, USA) was used in this research. It has a molecular weight of 72.06 g/mol and the formula C_3_H_4_O_2_. According to the manufacturer, the tested PA is a liquid with color faint yellow to amber to brown, with the content of arsenic, cadmium, mercury, and lead below detection limit, ≤3 ppm, ≤1 ppm, ≤1 ppm, ≤10 ppm, respectively. Moreover, the pyroligneous acid purity as acetic acid at 4.5–6.5% was determined by NaOH titration, while the other organic components were not mentioned by the producer of this substance. It appears as an amber-coloured liquid with a characteristic smoky smell. Before preparing all PA concentrations, pure PA was filtered through a 0.2 µm filter, and subsequent dilutions (Figure S6) were prepared using demineralised water filtered through a 0.2 µm sterile filter and sterilised [36]. All these operations were performed inside a sterilised laminar flow cabinet with aspirated air. The pyroligneous acid was not sterilized because, during sterilization, the temperatures reach around 125 °C and can influence the molecular structure of the substances that give PA its antimicrobial and antifungal properties. Furthermore, we did not neutralize the pH of the pyroligneous acid, as the substance, if neutralized, only has effects on the growth of bacteria and not of fungi [36].

### 4.3. Inhibition Test

The research included the effectiveness of different concentrations of PA: PA 0, PA pure, PA 1:2, PA 1:8, PA 1:16, PA 1:32, and PA 1:36 against *B. cinerea*. For a preliminary analysis, we applied the inhibition test as in Frąc et al. [43] and Pylak et al. [44], with some modifications presented below. All the three strains of *B. cinerea* were pre-cultured in potato dextrose agar medium (PDA) (BioMaxima S.A., Lublin, Poland) to obtain enough mycelium for this analysis. After 5 days, the mycelium was collected, and after homogenisation inside the FF inoculating fluid (BiOLOG, Hayward, CA, USA), a transmittance of 75% was measured before spreading 300 µL on inoculum in each Petri dish. After the application of the fungal mycelium, three sterile paper discs were placed on the medium surface, and 15 µL of each different PA concentration was applied of the surface of the discs. The plates were incubated at 27 °C and every 24 h the diameter of the inhibition zone was measured. Each plate contained three paper discs with particular concentration of PA as the three replicates of the analysis. For the concentration PA 0, 15 µL of sterilised demineralised water was added instead of pyroligneous acid, while PA pure included concentrated PA.

### 4.4. MT2 Microplates Analysis

In order to analyse the ability of *B. cinerea* to decompose pyroligneous acid, MT2 microplates (BiOLOG, Hayward, CA, USA), previously used for fungal sensitivity evaluation against fungicides by Frąc et al. [43], were used. Three different fungal strains were cultivated in PDA (BioMaxima S.A., Lublin, Poland) and potato dextrose broth (PDB) (BioMaxima S.A., Lublin, Poland) media for 5 days and incubated at 27 °C. The mycelium from both media was collected, and the mycelium from PDA (BioMaxima S.A., Lublin, Poland) was homogenised inside the FF inoculating fluid (BiOLOG, Hayward, CA, USA), whereas the mycelium from PDB was homogenised inside the PDB medium (BioMaxima S.A., Lublin, Poland). The transmittances of both prepared inocula were adjusted to 75%. Into each well, 50 µL of inoculum was added, and then 100 µL of each of the 6 different concentration (PA 0, PA pure, PA 1:2, PA 1:8, PA 1:16, PA 1:32, and PA 1:36) was incorporated. For the concentration PA 0, 100 µL of sterilised demineralised water was added instead of PA. Furthermore, a control treatment, composed of 50 µL of the FF inoculating fluid (BiOLOG, Hayward, CA, USA) or PDB medium (BioMaxima S.A., Lublin, Poland) plus 100 µL of sterilised demineralised water was prepared. In each plate, three replications for each treatment were prepared. The scheme of the preparation of the MT2 microplates (BiOLOG, Hayward, CA, USA) is shown in Figure S7. All the plates were incubated at 27 °C for 10 days, and, every 24 h, the optical density at 490 nm (to observe the absorption of the nutrients) and 750 nm (to detect the fungal biomass production) through the BIOLOG Microstation (BiOLOG, Hayward, CA, USA) were measured. From all the results we subtracted the values of the respective optical densities of all analysed PA concentrations without the addition of *Botrytis cinerea* mycelium.

### 4.5. FF Microplates Analysis

The phenotypic analysis was made through the FF microplate (BiOLOG, Hayward, CA, USA) and was prepared following the protocol of Frąc [45]. The FF microplates (BiOLOG, Hayward, CA, USA) are composed from 95 different carbon sources. For this experiment, three concentration of PA from the previous analysis were selected, including PA 1:8, PA 1:16, and PA 1:36. The reasons for selecting these concentrations included the need for a sufficient amount of mycelium to grow within these three different concentrations of pyroligneous acid in order to prepare three plates for each analysed concentration. Due to the fact that *B. cinerea* growth was completely inhibited on selected PA concentrations (Figure S8), we decided to perform additional optimization with the PA concentrations PA 0, PA 1:400, PA 1:800, and PA 1:1600. The three fungal stains were pre-cultured in PDB (BioMaxima S.A., Lublin, Poland) medium with particular concentrations of PA. After 5 days of incubation at 27 °C, the mycelium was collected and 75% of transmittance of inoculum was adjusted inside the FF inoculating fluid (BiOLOG, Hayward, CA, USA). 100 µL inoculum was added into each well and three replicates for each treatment were performed. All the microplates were incubated at 27 °C, and, every 24 h, the plates were recorded through the BIOLOG Microstation (BiOLOG, Hayward, CA, USA). The results of the substrate utilisation (at 490 nm) and the biomass production (at 750 nm) were collected during 10 incubation days. The Average Well Colour Development (AWCD) index, Average Well Density Development (AWDD) index, and substrate richness were calculated. AWCD and AWDD were calculated by adding the respective optical densities at 490 nm and 750 nm (corrected with the respective water value), all divided by 95 (which is the number of analysed resources in each plate). Substrate richness (R) was calculated by identifying how many substrates recorded an optical density greater than 0.25 [43,46].

### 4.6. Statistical Analysis

The inhibition test results were presented through a heatmap. Firstly, all the measurements were rearranged in a range between 0 and 1 and then a Euclidean distance matrix and the Ward method were applied to obtain the heatmaps and the cluster analysis, respectively. The results from the MT2 microplates (BiOLOG, Hayward, CA, USA) were grouped in three different groups: low, medium, medium, and fast. To all the results obtained from the MT2 and the FF microplates (BiOLOG, Hayward, CA, USA) we applied the Kruskal–Wallis test and the post hoc Wilcox test to observe the results that were statistically different. For all these analyses we used the R software v 4.1.3 (packages: *ComplexHeatmap*, *circlize*, *caret*, *Hmisc*) (https://www.r-project.org/index.html, accessed on 30 December 2022).

## 5. Conclusions

Overall, all of these analyses provide a picture of the effects of pyroligneous acid on the phytopathogenic fungus *Botrytis cinerea*.

The results indicated the inhibiting effect of PA on the growth of the phytopathogen *B. cinerea*. It was found that the *B. cinerea* fungus is sensitive to different concentrations of pyroligneous acid, even under low concentrations of the PA.

Pyroligneous acid caused metabolic stress for *B. cinerea* fungal strains and affected the AWCD, AWDD and R biodiversity indices of tested fungi. 

The results confirm that metabolic research using MT2 and FF microplates is very useful in the determination of efficiency of PA against phytopathogens.

As biodiversity indices showed significant differences in metabolic profiles of *B. cinerea* under the influence of PA, future research should focus on deeper analyses, in particular, carbon source compounds and groups, in order to indicate the compounds most responsible for the inhibition effect of PA. 

## Data Availability

The data presented in this study are available on request from the corresponding author.

## Supplementary Materials

The supporting information can be downloaded at https://www.mdpi.com/article/10.3390/ijms24043080/s1.
