# Supplementary material for: The Antifungal Effect of Pyroligneous Acid on the Phytopathogenic Fungus Botrytis cinerea
Source: Int J Mol Sci. 2023 Feb 4;24(4):3080. doi: 10.3390/ijms24043080 (PMC9959546; doi:10.3390/ijms24043080)
Supplement: Supplementary file 1 [file ijms-24-03080-s001.zip › ijms-2164633-supplementary.pdf]

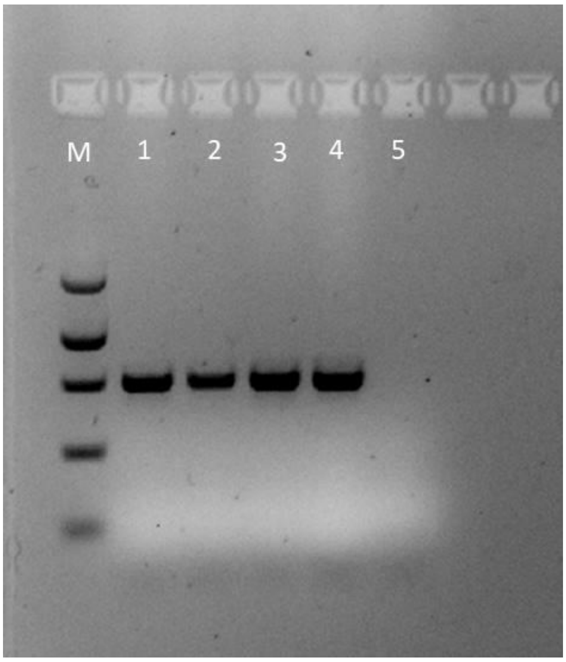

Figure S1. Gel electrophoresis of the PCR analysis with a specific primer for the detection of the fungus *Botrytis cinerea* (Patent number P.431989). This primer product a PCR-product of 406 bp. M: marker 50-1000 bp, 1: G275/18, 2: G276/18, 3: G323/18, 4: G3/19, 5: NTC (No Template Control).

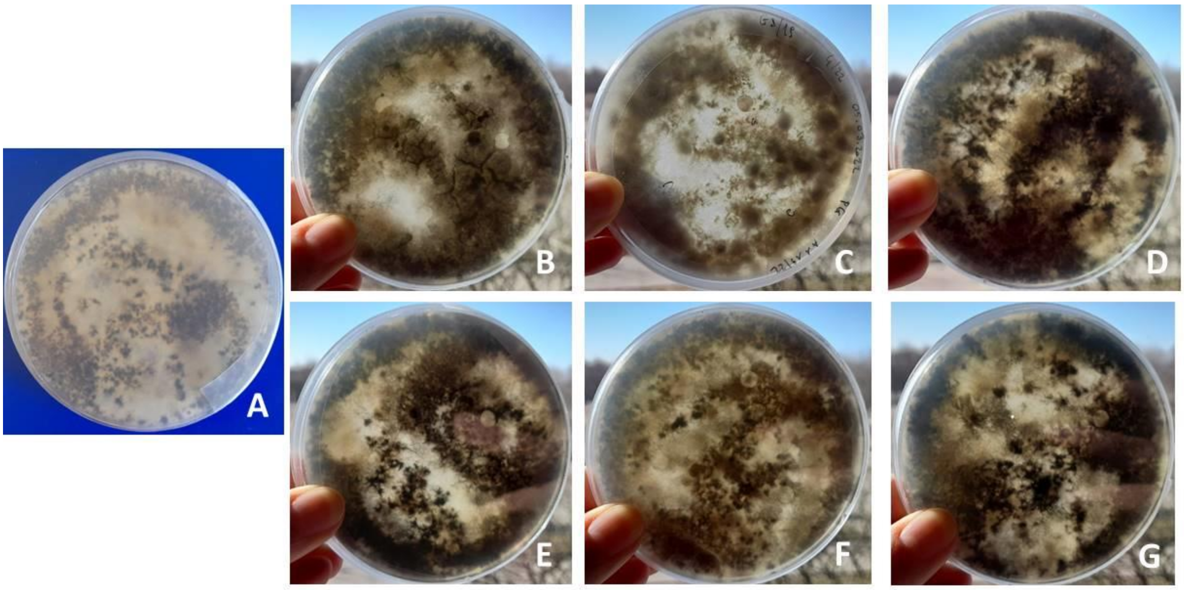

Figure S2. Photos of the inhibition test of Botrytis cinerea after 5 days of the incubation at 27°C after the adding of difference concentration of pyroligneous acid (PA). Each photo represent the fungus incubated at different dilutions of PA: PA 0 (A), PA pure (not diluted) (B), PA 1:2 (C), PA 1:8 (D), PA 1:16 (E), PA 1:32 (F), and PA 1:36 (G).

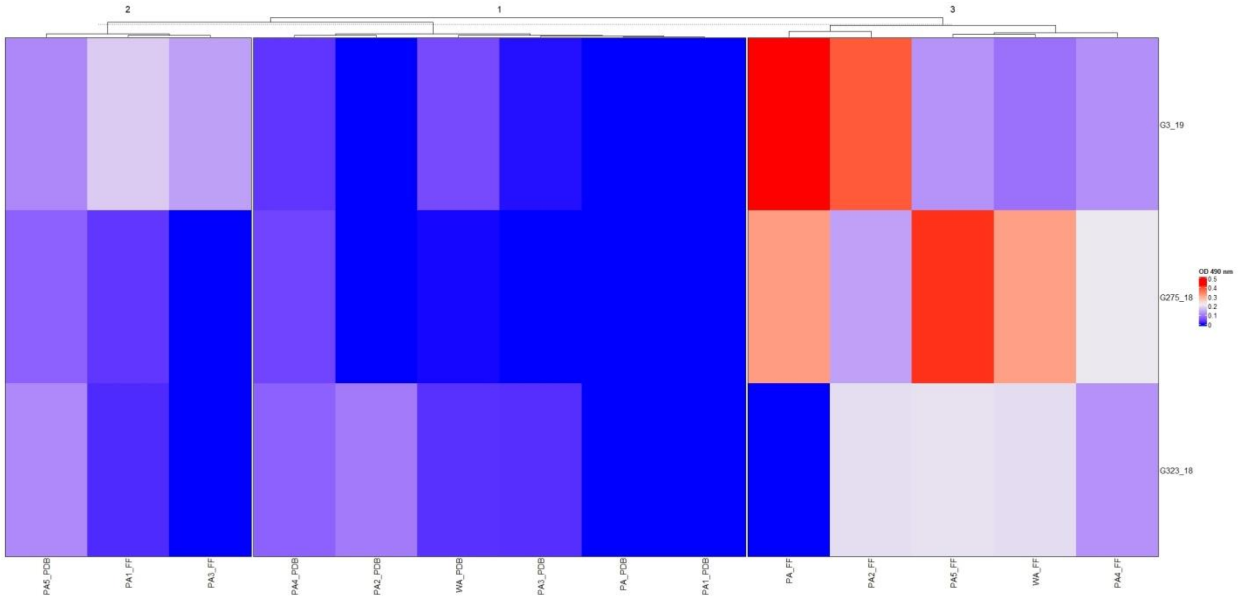

Figure S3. Heatmap of the fungal respiration activity (optical density 490 nm). We represented the graphs in function of the Euclidean distance matrix and the cluster analysis was made through the Ward method. Abbreviation: WA is without adding PA, PA is PA pure, PA1 is PA 1:2, PA2 is PA 1:8, PA3 is PA 1:16, PA4 is PA 1:32, and PA5 is PA 1:36.

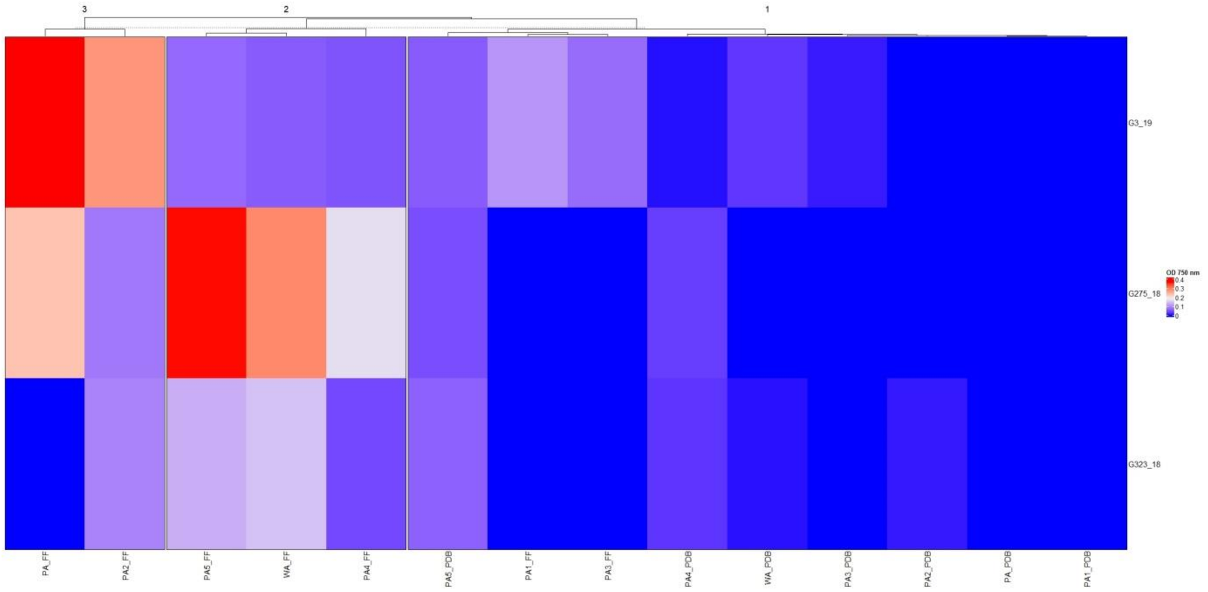

Figure S4. Heatmap of the fungal biomass production (optical density 750 nm). We represented the graphs in function of the Euclidean distance matrix and the cluster analysis was made through the Ward method. Abbreviation: WA is without adding PA, PA is PA pure, PA1 is PA 1:2, PA2 is PA 1:8, PA3 is PA 1:16, PA4 is PA 1:32, and PA5 is PA 1:36.

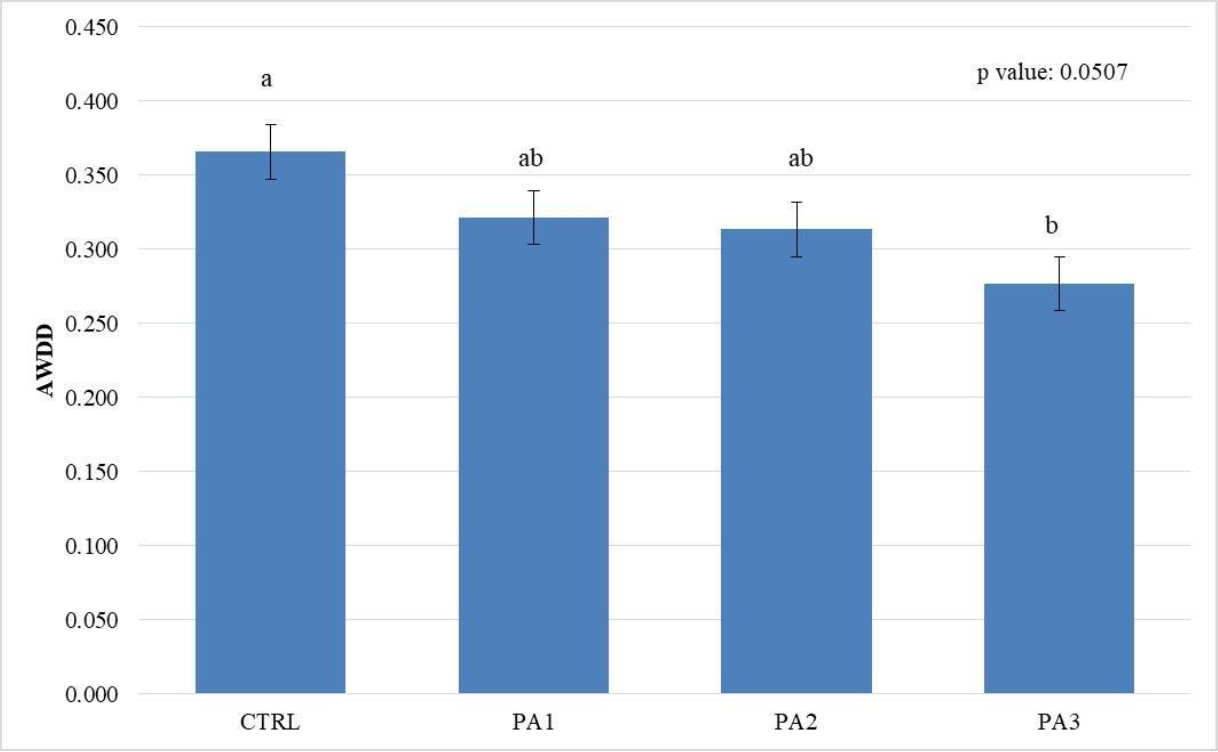

Figure S5. AWDD index for the four different analysed treatments. Each values is represented with the standard error. The lower case letters represents the results obtained after applying the post hoc Wilcox test (Table S20). Abbreviation: Control is without adding PA (PA 0), PA1 (PA 1:400), PA2 (PA 1:800), PA3 (PA 1:1600).

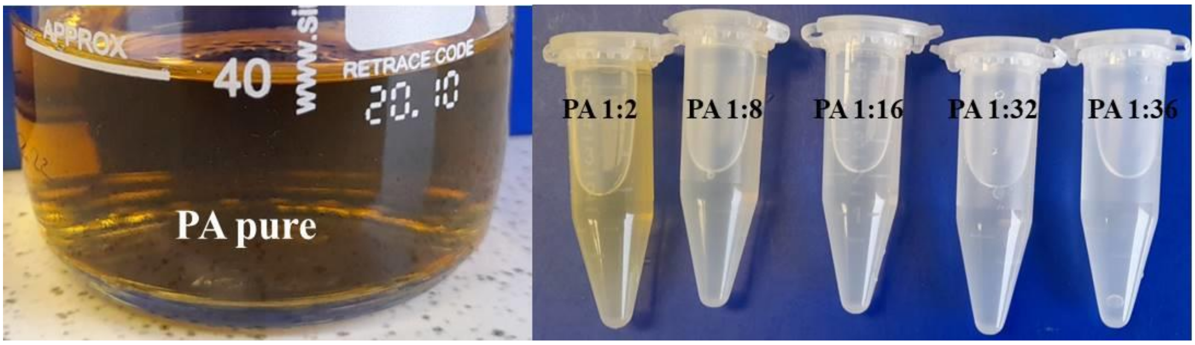

Figure S6. Pyroligneous acid and its dilutions that we prepared with filtered acid (by 0.2 µm filter) and sterilised demineralised water in sterile condition.

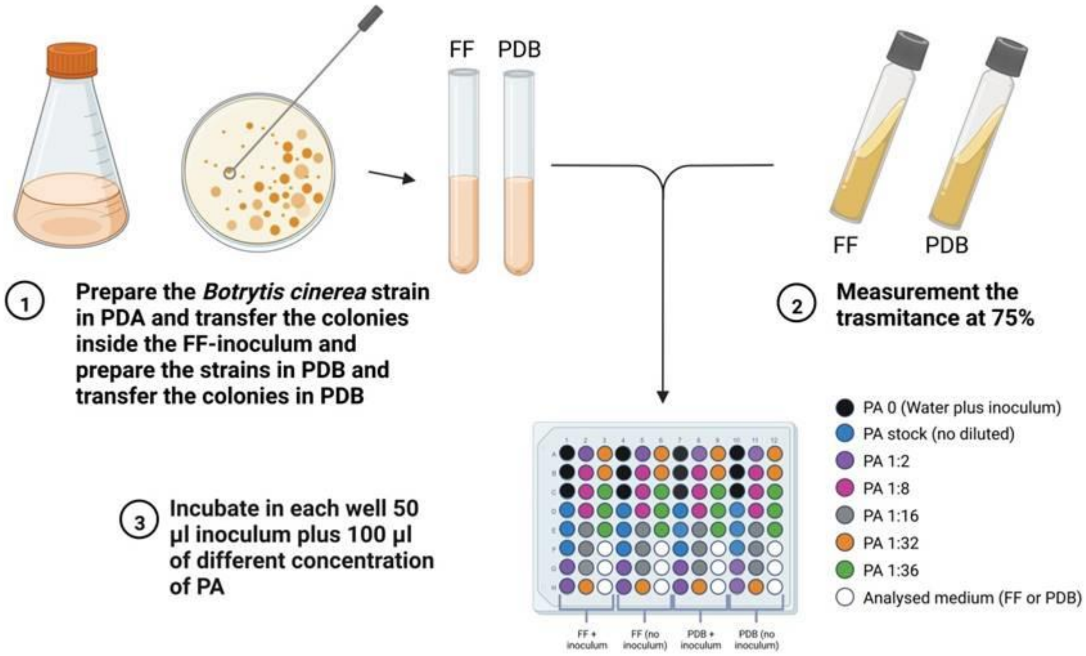

Figure S7. Scheme representation of the MT2 microplate preparation. Inside each plate there is three replication for each analysed treatment. We prepared in total three plate, one for each strain of *Botrytis cinerea*. Created with BioRender.com.

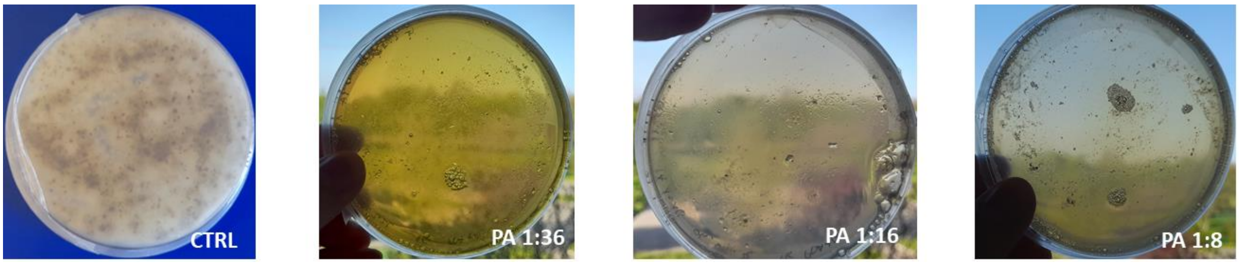

Figure S8. The photo of the Petri discs after the incubation of *Botrytis cinerea* at 27°C after 5 days.

Table S1. Kruskal-Wallis test results for the variable optical density 490 nm inside the FF inoculum for the MT2 microplate.

Effect	chi square test	df	p value
**Treatment**	137.1	6	**< 2.2e-16**
**Strain**	38.148	2	**5.204e-09**

Table S2. Post hoc Wilcox test results between optical density 490 nm and *Botrytis cinerea* strains from MT2 microplates incubated in FF inoculum results (p value 5.204e-09).

	**G275/18**	**G3/19**
**G3/19**	1.00	
**G323/18**	**9.6e-06**	**5.9e-09**

P value adjustment method: bonferroni.

Table S3. Post hoc Wilcox test results between optical density 490 nm and treatments from MT2 microplates incubated in FF inoculum results (p value < 2.2e-16).

	**Control**	**PA3**	**PA1**	**PA4**	**PA5**	**PA2**
**PA3**	**1.6e-14**					
**PA1**	**2.0e-06**	0.0779				
**PA4**	1.0000	**1.5e-08**	**0.0127**			
**PA5**	1.0000	**6.3e-15**	**2.2e-07**	0.8910		
**PA2**	0.4237	**< 2e-16**	**6.7e-11**	**0.0021**	1.0000	
**PA**	1.0000	**5.4e-10**	**0.0002**	0.6333	1.0000	1.0000

P value adjustment method: bonferroni.

Abbreviation: Control is without adding PA, PA is PA pure, PA1 is PA 1:2, PA2 is PA 1:8, PA3 is PA 1:16, PA4 is PA 1:32, and PA5 is PA 1:36.

Table S4. Kruskal-Wallis test results for the variable optical density 750 nm inside the FF inoculum for the MT2 microplate.

Effect	chi square test	df	p value
**Treatment**	132.58	6	**< 2.2e-16**
**Strain**	58.83	2	**1.68e-13**

Table S5. Post hoc Wilcox test results between optical density 750 nm and *Botrytis cinerea* strains from MT2 microplates incubated in FF inoculum results (p value 1.68e-13).

	**G275/18**	**G3/19**
**G3/19**	1.00	
**G323/18**	**2.3e-09**	**1.3e-12**

P value adjustment method: bonferroni.

Table S6. Post hoc Wilcox test results between optical density 750 nm and treatment from MT2 microplates incubated in FF inoculum results (p value < 2.2e-16).

	Control	PA3	PA1	PA4	PA5	PA2
**PA3**	**< 2e-16**					
**PA1**	**1.5e-09**	1.00000				
**PA4**	0.14065	**1.2e-08**	**0.00194**			
**PA5**	1.00000	**7.7e-14**	**4.0e-07**	0.82566		
**PA2**	1.00000	**< 2e-16**	**3.4e-10**	0.16104	1.00000	
**PA**	1.00000	**8.1e-09**	**0.00019**	1.00000	1.00000	1.00000

P value adjustment method: bonferroni.

Abbreviation: Control is without adding PA, PA is PA pure, PA1 is PA 1:2, PA2 is PA 1:8, PA3 is PA 1:16, PA4 is PA 1:32, and PA5 is PA 1:36.

Table S7. Kruskal-Wallis test results for the variable optical density 490 nm inside the PDB inoculum for the MT2 microplate.

Effect	chi square test	df	p value
**Treatment**	277.13	6	**< 2.2e-16**
**Strain**	30.725	2	**2.129e-07**

Table S8. Post hoc Wilcox test results between optical density 490 nm and *Botrytis cinerea* strains from MT2 microplates incubated in PDB medium results (p value 2.129e-07).

	**G275/18**	**G3/19**
**G3/19**	0.90742	
**G323/18**	**1.6e-07**	**0.00037**

P value adjustment method: bonferroni.

Table S9. Post hoc Wilcox test results between optical density 490 nm and treatment from MT2 microplates incubated in PDB medium results (p value < 2.2e-16).

	Control	PA3	PA1	PA4	PA5	PA2
**PA3**	1.00000					
**PA1**	**7.0e-16**	**8.0e-11**				
**PA4**	**0.02484**	**0.00030**	**< 2e-16**			
**PA5**	**1.2e-12**	**3.7e-15**	**< 2e-16**	**3.0e-09**		
**PA2**	1.00000	1.00000	**7.8e-07**	**0.00021**	**7.3e-10**	
**PA**	**1.9e-11**	**1.6e-07**	1.00000	**< 2e-16**	**< 2e-16**	**0.00020**

P value adjustment method: bonferroni.

Abbreviation: Control is without adding PA, PA is PA pure, PA1 is PA 1:2, PA2 is PA 1:8, PA3 is PA 1:16, PA4 is PA 1:32, and PA5 is PA 1:36.

Table S10. Kruskal-Wallis test results for the variable optical density 750 nm inside the PDB inoculum for the MT2 microplate.

Effect	chi square test	df	p value
**Treatment**	227.24	6	**< 2.2e-16**
**Strain**	10.794	2	**0.004529**

Table S11. Post hoc Wilcox test results between optical density 750 nm and *Botrytis cinerea* strains from MT2 microplates incubated in PDB medium results (p value 0.004529).

	**G275/18**	**G3/19**
**G3/19**	1.0000	
**G323/18**	**0.0038**	**0.0682**

P value adjustment method: bonferroni.

Table S12. Post hoc Wilcox test results between optical density 750 nm and treatment from MT2 microplates incubated in PDB medium results (p value < 2.2e-16).

	Control	PA3	PA1	PA4	PA5	PA2
**PA3**	1.00000					
**PA1**	**1.5e-13**	**4.7e-08**				
**PA4**	1.00000	0.60126	**6.1e-15**			
**PA5**	**1.5e-10**	**5.2e-12**	**< 2e-16**	**1.7e-07**		
**PA2**	0.09204	1.00000	**6.3e-08**	**0.00973**	**< 2e-16**	
**PA**	**1.3e-09**	3.7e-05	1.00000	**3.4e-11**	**< 2e-16**	**0.00015**

P value adjustment method: bonferroni.

Abbreviation: Control is without adding PA, PA is PA pure, PA1 is PA 1:2, PA2 is PA 1:8, PA3 is PA 1:16, PA4 is PA 1:32, and PA5 is PA 1:36.

Table S13. Grouping value of MT2 microplate in three different group.

Strain	Inoculum	Treatment	OD 490 nm*		OD 750 nm*		Ratio 490/750**	
G3/19	FF	PA	**0.427**	4	**0.365**	4	**1.172**	2
G275/18	FF	PA	**0.313**	4	**0.228**	4	1.372	3
G323/18	FF	PA	0.000	1	0.000	1	0.000	1
G3/19	FF	PA1	**0.179**	4	**0.107**	4	1.678	3
G275/18	FF	PA1	0.034	2	0.000	1	NA	NA
G323/18	FF	PA1	0.023	2	0.000	1	NA	NA
G3/19	FF	PA2	**0.382**	4	**0.274**	4	1.392	3
G275/18	FF	PA2	**0.137**	4	**0.081**	4	1.683	3
G323/18	FF	PA2	**0.197**	4	**0.091**	4	2.173	4
G3/19	FF	PA3	0.135	3	0.071	3	1.899	4
G275/18	FF	PA3	0.000	1	0.000	1	0.000	1
G323/18	FF	PA3	0.000	1	0.000	1	0.000	1
G3/19	FF	PA4	0.118	3	0.051	3	2.327	4
G275/18	FF	PA4	**0.209**	4	**0.169**	4	**1.236**	2
G323/18	FF	PA4	0.120	3	0.042	3	2.880	4
G3/19	FF	PA5	0.122	3	0.068	3	1.792	3
G275/18	FF	PA5	**0.411**	4	**0.363**	4	**1.132**	2
G323/18	FF	PA5	**0.201**	4	**0.127**	4	1.574	3
G3/19	PDB	PA	0.000	1	0.000	1	0.000	1
G275/18	PDB	PA	0.000	1	0.000	1	0.000	1
G323/18	PDB	PA	0.000	1	0.000	1	0.000	1
G3/19	PDB	PA1	0.000	1	0.000	1	0.000	1
G275/18	PDB	PA1	0.000	1	0.000	1	0.000	1

G323/18	PDB	PA1	0.000	1	0.000	1	0.000	1
G3/19	PDB	PA2	0.000	1	0.000	1	0.000	1
G275/18	PDB	PA2	0.000	1	0.000	1	0.000	1
G323/18	PDB	PA2	0.098	3	0.009	2	11.195	4
G3/19	PDB	PA3	0.006	2	0.010	2	**0.536**	2
G275/18	PDB	PA3	0.000	1	0.000	1	0.000	1
G323/18	PDB	PA3	0.026	2	0.000	1	NA	NA
G3/19	PDB	PA4	0.032	2	0.005	2	6.821	4
G275/18	PDB	PA4	0.045	2	0.034	3	**1.320**	2
G323/18	PDB	PA4	0.073	3	0.026	3	2.768	4
G3/19	PDB	PA5	0.112	3	0.057	3	1.949	4
G275/18	PDB	PA5	0.072	3	0.046	3	1.582	3
G323/18	PDB	PA5	0.113	3	0.061	3	1.835	3

* The groups 1,2,3,4 mean slow, slow-medium, fast-medium, fast.

** The groups 1,2,3,4 mean efficient metabolism, medium metabolism, medium metabolism, inefficient metabolism.

Abbreviation: PA is PA pure, PA1 is PA 1:2, PA2 is PA 1:8, PA3 is PA 1:16, PA4 is PA 1:32, PA5 is PA 1:36, and NA means not available.

Table S14. Kruskal-Wallis test results for the variable AWCD index.

Effect	chi square test	df	p value
**Treatment**	3.9353	3	0.2685
**Strain**	25.942	2	**2.32e-06**

Table S15. Post hoc Wilcox test results between AWCD index and *Botrytis cinerea* strains from FF microplates results (p value 2.326e-06).

	**G275/18**	**G3/19**
**G3/19**	**0.0017**	
**G323/18**	**1.8e-05**	**0.0202**

P value adjustment method: bonferroni.

Table S16. Kruskal-Wallis test results for the variable Substance richness (R) index for the optical density 490 nm.

Effect	chi square test	df	p value
**Treatment**	8.5862	3	**0.03533**
**Strain**	9.8374	2	**0.007309**

Table S17. Post hoc Wilcox test results between substance richness (R) index for optical density at 490 nm and treatment from FF microplates results (p value 0.03533).

	**Control**	**PA1**	**PA2**
**PA1**	0.141		
**PA2**	0.094	1.000	
**PA3**	1.000	0.852	0.537

P value adjustment method: bonferroni.

Abbreviation: Control is without adding PA (PA 0), PA1 (PA 1:400), PA2 (PA 1:800), PA3 (PA 1:1600).

Table S18. Post hoc Wilcox test results between substance richness (R) index for optical density at 490 nm and *Botrytis cinerea* strains from FF microplates results (p value 0.007309).

	**G275/18**	**G3/19**
**G3/19**	0.088	
**G323/18**	**0.008**	0.991

P value adjustment method: bonferroni.

Table S19. Kruskal-Wallis test results for the variable AWDD index.

Effect	chi square test	df	p value
**Treatment**	7.7838	3	0.0507
**Strain**	24.137	2	**5.738e-06**

Table S20. Post hoc Wilcox test results between AWDD index and treatment from FF microplates results (p value 0.0507).

	Control	PA1	PA2
**PA1**	0.762		
**PA2**	0.828	1.000	
**PA3**	**0.041**	0.989	1.000

P value adjustment method: bonferroni.

Abbreviation: Control is without adding PA (PA 0), PA1 (PA 1:400), PA2 (PA 1:800), PA3 (PA 1:1600).

Table S21. Post hoc Wilcox test results between AWDD index and *Botrytis cinerea* strains from FF microplates results (p value 5.738e-06).

	**G275/18**	**G3/19**
**G3/19**	**0.024**	
**G323/18**	**3e-05**	**0.004**

P value adjustment method: bonferroni.

Table S22. Kruskal-Wallis test results for the variable Substance richness (R) index for the optical density 750 nm.

Effect	chi square test	df	p value
**Treatment**	5.6705	3	0.1288
**Strain**	8.9368	2	**0.01147**

Table S23. Post hoc Wilcox test results between substance richness (R) index for optical density at 750 nm and *Botrytis cinerea* strains from FF microplates results (p value 0.01147).

	**G275/18**	**G3/19**
**G3/19**	0.142	
**G323/18**	**0.013**	0.724
